# We Must Find the Problem in the Intrinsic Mechanisms of Both Media and Medical Research

**DOI:** 10.1371/journal.pmed.0020364

**Published:** 2005-10-25

**Authors:** Luciano Benedetti

**Affiliations:** **1**Saatchi and Saatchi Health CareMilanoItaly

Desmond Gale is right: the media don't care about health promotion and disease prevention [1,2]. In fact, this is not their job; the main task of the media is to inform the public correctly. To inform, especially about medicine and health, means to disseminate all relevant news, together with (and never without) all information needed to understand the facts and to use them.

I'm very concerned about the present situation of the medical media. I see three different problems. Firstly, journalists often fail to carry out this main task because they are forced by the media industry to produce spectacle or to entertain their public. Secondly, many journalists don't know enough about medicine, and many scientists are unable, or refuse, to communicate. Some scientists, in contrast, use the media as a personal launch window. Thirdly, with the increasing privatization of research funding, many clinical studies are classified (that is, the results, if negative, are kept secret by the sponsor) and often full of methodological bias. On the other hand, a lot of scientific communication is masked advertising.

These factors make it difficult to achieve good health journalism. Fortunately, many colleagues all over the world (both journalists and researchers) are trying to find solutions to these problems.
